# Effectiveness of early ureteric stenting for urosepsis associated with urinary tract calculi

**DOI:** 10.1186/cc11981

**Published:** 2013-03-19

**Authors:** S Nishiguchi, Y Tokuda

**Affiliations:** 1 Shonan Kamakura General Hospital, Kamakura, Japan; 2 Mito Kyodo General Hospital, Mito, Japan

## Introduction

Urosepsis associated with urinary tract calculi is a critical disease. Patients with this condition occasionally require drainage, mostly ureteric stent placement and these patients need longer hospitalization. However, indications for timely ureteric stenting for urosepsis associated with urinary tract calculi have not been clearly determined. The objective of the study was to evaluate whether earlier stent placement might lead to shorter length of stay (LOS) in hospitals.

## Methods

For patients who required ureteric stent procedures for urosepsis associated with urinary tract calculi in our hospital from July 2008 to July 2012, we compared the LOS in our hospital between patients with earlier stenting and those without it. A linear regression model was used for multivariable-adjusted comparison.

## Results

In a total of 30 patients (mean age, 72; 13 males), the mean days from emergency room admission to ureteric stenting was 3.53days (range, 1 to 14 days) and the overall mean LOS was 36 days (range, 8 to 102 days). The early-stenting group (mean LOS, 21 days) had significantly shorter LOS than the delayed-stenting group (mean LOS, 50 days) with adjusted β coefficient of -26 days (95% CI, -46, -6).

## Conclusion

In patients with urosepsis associated with urinary tract calculi, earlier stenting within 2 days of admission may reduce the hospital LOS.
